# Extending the Inhibition Profiles of Coumarin-Based Compounds Against Human Carbonic Anhydrases: Synthesis, Biological, and In Silico Evaluation

**DOI:** 10.3390/molecules24193580

**Published:** 2019-10-04

**Authors:** Victor Kartsev, Athina Geronikaki, Silvia Bua, Alessio Nocentini, Anthi Petrou, Boris Lichitsky, Mykhaylo Frasinyuk, Janis Leitans, Andris Kazaks, Kaspars Tars, Claudiu T. Supuran

**Affiliations:** 1 InterBioscreen, 119019 Moscow, Russia; vkartsev@ibscreen.chg.ru; 2 School of Health, Department of Pharmacy, Aristotle University of Thessaloniki, 54124 Thessaloniki, Greece; anthi.petrou.thessaloniki1@gmail.com; 3 Dipartimento Neurofarba, Sezione Di Scienze Farmaceutiche e Nutraceutiche, Universita’ Degli Studi Di Firenze, Sesto Fiorentino, 50019 Florence, Italy; silvia.bua@unifi.it (S.B.); alessio.nocentini@unifi.it (A.N.); 4 Zelinsky Institute of Organic Chemistry, Leninsky prospect, 119991 Moscow, Russia; blich2006@mail.ru; 5 Institute of Bioorganic Chemistry and Petrochemistry, National Academy of Science of Ukraine, 02094 Kiev, Ukraine; mykhaylo.frasinyuk@ukr.net; 6 Latvian Biomedical Research and Study Center, Ratsupites 1, Riga LV-1067, Latvia; janis.leitans@biomed.lu.lv (J.L.); andris@biomed.lu.lv (A.K.); kaspars@biomed.lu.lv (K.T.)

**Keywords:** carbonic anhydrase, tumor-associated isoform, prodrug, coumarin, docking, selectivity

## Abstract

Carbonic anhydrases (CAs, EC 4.2.1.1) catalyze the fundamental reaction of CO_2_ hydration in all living organisms and are actively involved in the regulation of a plethora of pathological and physiological conditions. A set of new coumarin/ dihydrocoumarin derivatives was here synthesized, characterized, and tested as human CA inhibitors. Their inhibitory activity was evaluated against the cytosolic human isoforms hCA I and II and the transmembrane hCA IX and hCA XII. Two compounds showed potent inhibitory activity against hCA IX, being more active or equipotent with the reference drug acetazolamide. Computational procedures were used to investigate the binding mode of this class of compounds within the active site of hCA IX and XII that are validated as anti-tumor targets.

## 1. Introduction

Coumarins are naturally occurring compounds and their richest sources are higher plants of *Rutaceae* and *Umbelliferae* types [1]. Coumarin is known to be as multitarget pharmacophore with wide variety of biological activities and, therefore, attracts the interest of the scientific community. In fact, coumarin derivatives were shown to act as an anticoagulant [2], anticancer [3,4,5], anti-inflammatory [6,7,8], antimicrobial [9,10], antifungal [11,12], antidiabetic [13], anti-viral [14], anti-Alzheimer [15,16,17], MAO-inhibitor [18], antioxidant [19,20], antihyperlipidemic [21], and other biological activities also identified [22,23,24,25,26]. A plethora of publications refers to carbonic anhydrase (CAs, EC 4.2.1.1) inhibitory activity of coumarin derivatives [27,28,29,30]. CAs are ubiquitous metalloenzymes in all life kingdoms [8,9]. They catalyze the reversible hydration of CO_2_ with formation of bicarbonate and protons, thus efficiently converting two neutral molecules in a weak base (bicarbonate) and a very strong acid (H^+^ ion). For this reason, in most organisms investigated so far, these enzymes are involved in pH regulation as well as several crucial metabolic pathways. At least seven distinct CA genetic families are known to date (α-, β-, γ-, ζ-, η-, and θ-CAs), and their diffusion and physiological roles have been investigated in details mainly in vertebrates, including humans, that only possess α-CAs, but with quite a large number of isoforms (15 CA isoforms are known in humans, hCA I-XIV, with two V-type ones, CA VA and VB) [8,9]. The CA inhibitors (CAIs) possess many pharmacologic applications, such as diuretics [31], anti-glaucoma, antiobesity, anti-tumor agent, and recently, anti-inflammatory and antineuropathic pain [32]. In this paper, we report synthesis, kinetic evaluation of the CA inhibitory activity, and in silico studies of a new set of coumarin-based derivatives, whose CA inhibitory scaffold was previously shown to act selectively against isoforms overexpressed in tumors that are as CA IX and XII [27,28,29,30,31,32].

## 2. Results and Discussion

### 2.1. Drug Design and Chemistry

Five CA inhibition mechanisms have been identified to date, but complete structural binding data are only available for four of them [33]. These are: (i) zinc binders; (ii) inhibitors anchoring to the zinc bound water/hydroxide ion; (iii) inhibitors occluding the entrance to the active site; (iv) inhibitors binding out of the active site; and (v) compounds with unknown inhibition mechanism.

The occlusion of the binding site entrance as a CA inhibition mechanism was evidenced for the first time with a natural product coumarin, isolated from the Australian plant Leionema ellipticum and, therefore, for the simple coumarin [27]. Successively, the antiepileptic drug lacosamide, 5- and 6-membered lactones and thiolactones or quinolinones were observed to possess significant CA inhibitory properties probably sharing a common mechanism of action [33]. In detail, X-ray crystallography studies were conducted, which showed that coumarins acts as prodrug - at least - in human CAs being hydrolyzed to the active species 2-hydroxycinnamic acids by the CA esterase activity [33]. The binding of the coumarin active species occurs in regions of the CA active site that most significantly differ among the various human isoforms known to date, furnishing the explanation for the high isoform-selective inhibitory profile shown by such a class of compounds [33].

To extend the structure-activity relationship of coumarins with hCAs, we report here the synthesis of a new set of coumarin-based derivatives to be screened for the inhibition of the ubiquitous hCA I and II and the tumor-associated hCA IX and XII. 

**Scheme 1:**
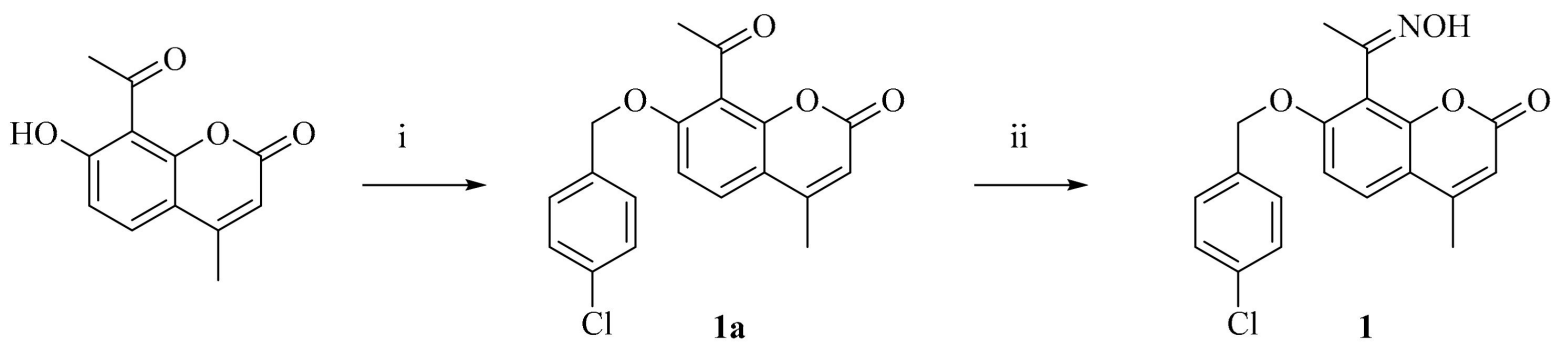
Synthesis of coumarin **1**. Reagent and conditions: (i) p-ClC_6_H_4_CH_2_Cl, K_2_CO_3_, acetone, reflux; (ii) NH_2_OH⋅HCl, AcONa, EtOH, reflux.

Oxime was synthesized by alkylation of 8-acetyl-4-methylumbelliferone with 4-chlorobenzyl chloride in dry acetone in presence of K_2_CO_3_. The formed 8-acylcoumarin **1a** was treated with hydroxylamine which afford target oxime **1** (Scheme 1).

**Scheme 2:**
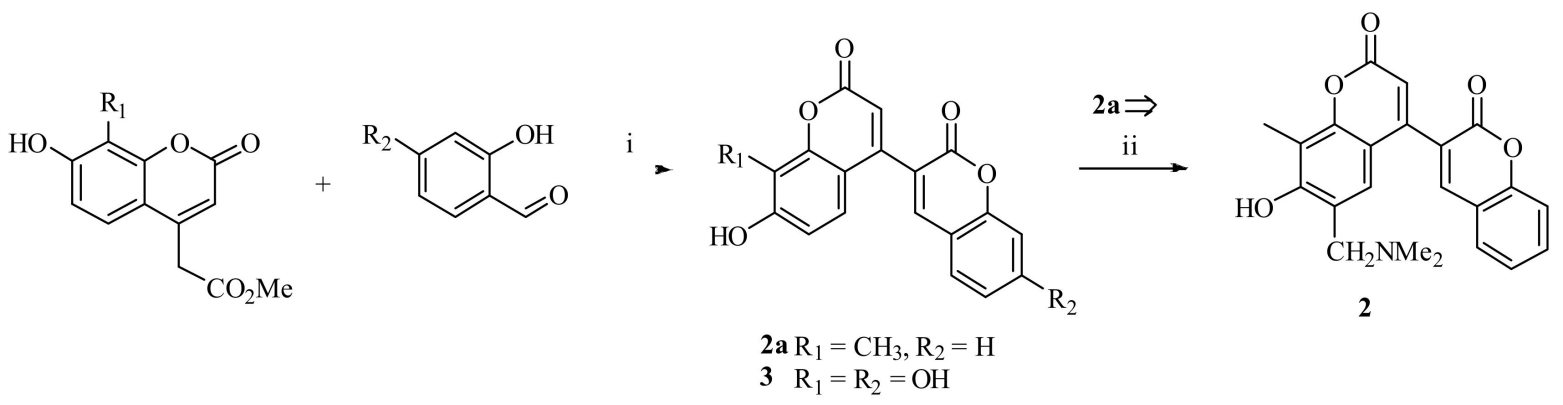
Synthesis of coumarins **2** and **3**. Reagent and conditions: (i) K_2_CO_3_, DBU, 1,4-dioxane, reflux; (ii) CH_2_(NMe_2_)_2_, 1,4-dioxane, reflux.

3,4’-Bicoumarins **2** and **3** were synthesized according to Scheme 2. Thus, heating of coumarin-4-acetic acid esters with variously substituted aldehydes led to one-pot formation of hydroxylated 3,4’-bicoumarins **2a** and **3**. Aminomethylation of coumarin **2a** with bisdimethylaminomethane in 1,4-dioxane affords aminomethylderivative **2** (Scheme 2).

**Scheme 3:**
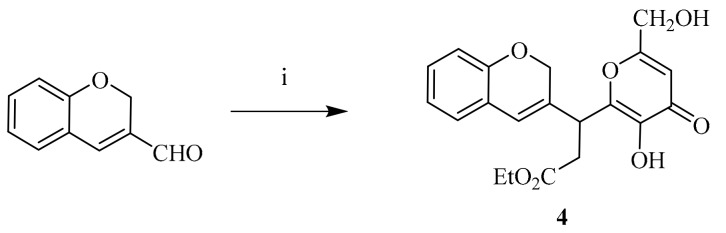
Synthesis of 2-H-chromene **4**. Reagent and conditions: (i) kojic acid, Meldrum’s acid, Et_3_N, EtOH, reflux.

Ethyl ester **4** was synthesized by multicomponent reaction of 3-formyl-2H-chromene with kojic acid and Meldrum’s acid (2,2-dimethyl-1,3-dioxane-4,6-dione) (Scheme 3).

**Scheme 4:**
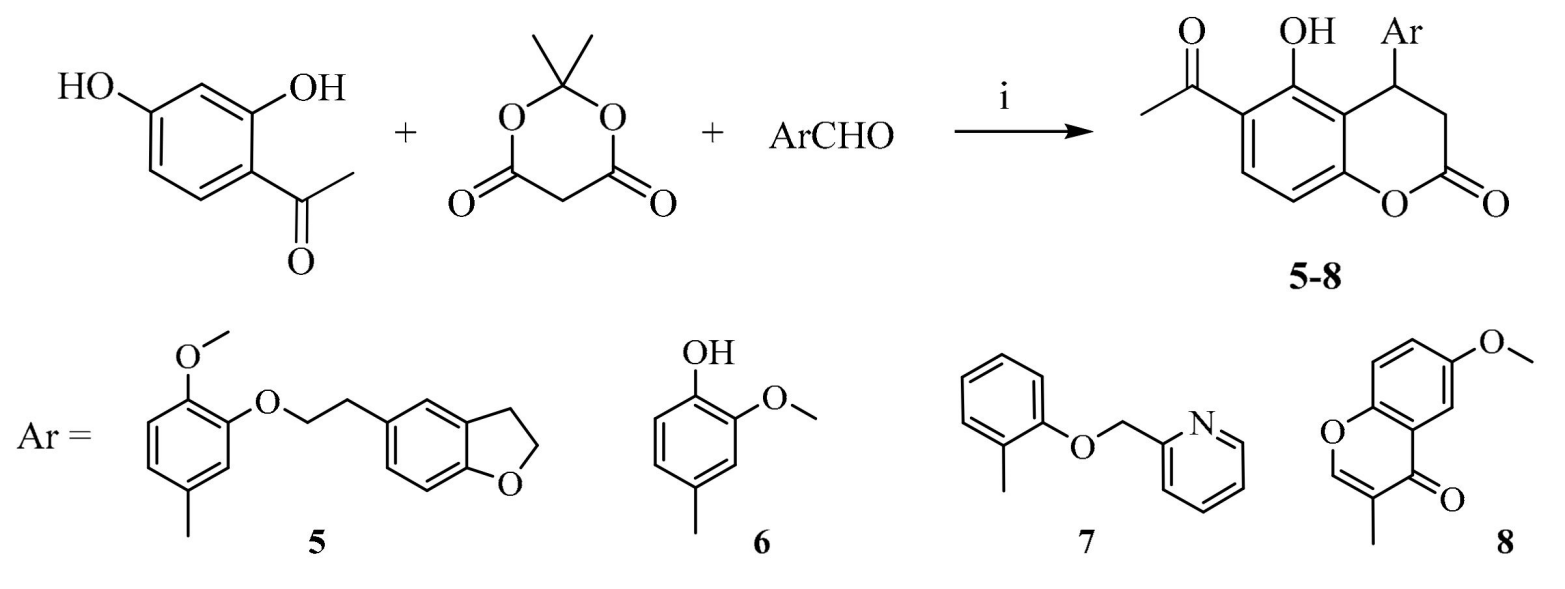
Synthesis of chroman-2-ones **5**–**8**. Reagent and conditions: (i) Et_3_N, MeOH, reflux.

Similar reaction of Meldrum’s acid with aromatic aldehydes and 2,4-dihydroxyacetophenone allows to synthesize 6-acetyl-4-aryl-5-hydroxy-3,4-dihydrocoumarins **5**–**8** (Scheme 4). All compounds were characterized by NMR spectra.

**Figure 1:**
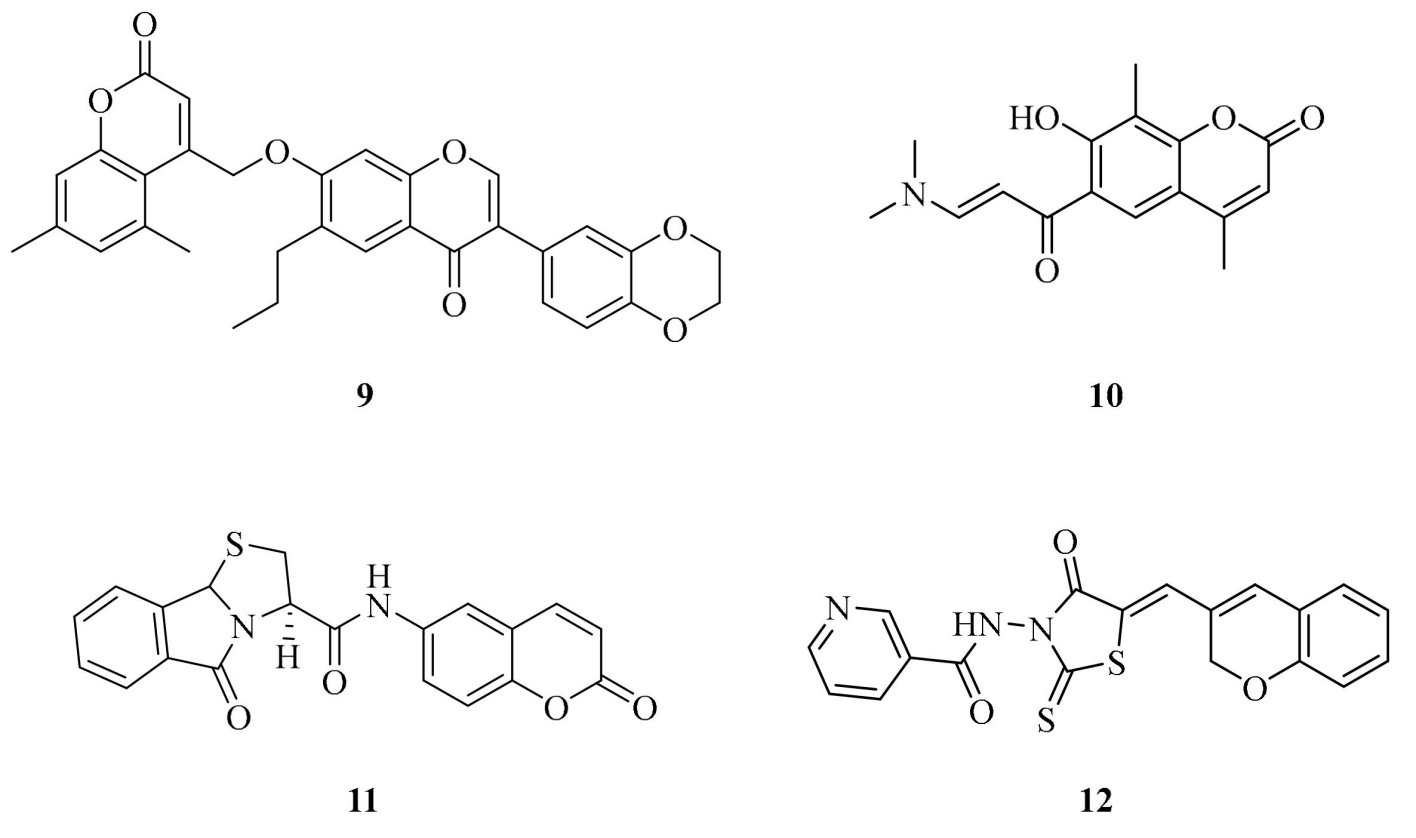
Structure of coumarin **9**–**11** and 2H-chromene **12**.

Coumarins **9** and **10** (Figure 1) were synthesized according to the literature procedures [34,35]. Compounds **11**, **12** (Figure 1) were purchased and included in the CA inhibition assays.

### 2.2. CA Inhibition

Coumarin-based compounds **1**–**12** were evaluated for their inhibition against the cytosolic CA I and II and the membrane-bound IX and XII by using a stopped-flow CO_2_ hydrase assay method. The clinically used acetazolamide (AAZ) was used as standard drug in the kinetic evaluation. The following SAR (this is not SAR but results of evaluation) can be worked out from the data reported in Table 1.

According to previously reported CA inhibition profiles of coumarin-based derivatives, none of **1**–**12** inhibited the ubiquitous and cytosolic hCA I and II below 10 μM. In contrast, the main tumor-associated isoform hCA IX was efficiently inhibited by derivatives **1**–**12** in a low to medium nanomolar range with inhibition constants (K_I_s) spanning between 9.4 and 243.1 nM. Not surprisingly, coumarin **11**, possessing the CA inhibitory scaffold less sterically hindered among the tested compounds, exhibited the best CA inhibitory action of the study with a K_I_ of 9.4 nM. For the same reason, coumarin **10** also acts as an equipotent CAI with the standard drug **AAZ** (K_I_ of 25.7 nM). Quite unexpectedly because of the steric hindrance produced by the oxime moiety in position 8, coumarin **1** showed a CA IX inhibitory efficacy (K_I_ of 49.3 nM) which is 2- to 5-fold greater than the remaining compounds. The latter’s show rather comparable K_I_s which range from 85.6 to 243.1 nM. 

A peculiar inhibition profile was instead measured for the other tumor-associated isoform hCA XII. In fact, unpredictably, most derivatives do not inhibit hCA XII up to 10 μM, whereas the subset composed by **1**, **4**, **10**–**12** shows a medium nanomolar inhibition against the isozyme with K_I_s in the range 432.1–603.8 nM. This might be due to the significant steric hindrance bore by most derivatives on the coumarin or coumarin-like scaffold.

### 2.3. Docking Studies

To rationalize the CA inhibitory profiles of Table 1, docking studies were undertaken with all assayed compounds in the active site of hCA IX and XII. The docking scores of all compounds and their hydrolyzed species in complex with CA IX and XII are shown in Table 2.

**Figure 2:**
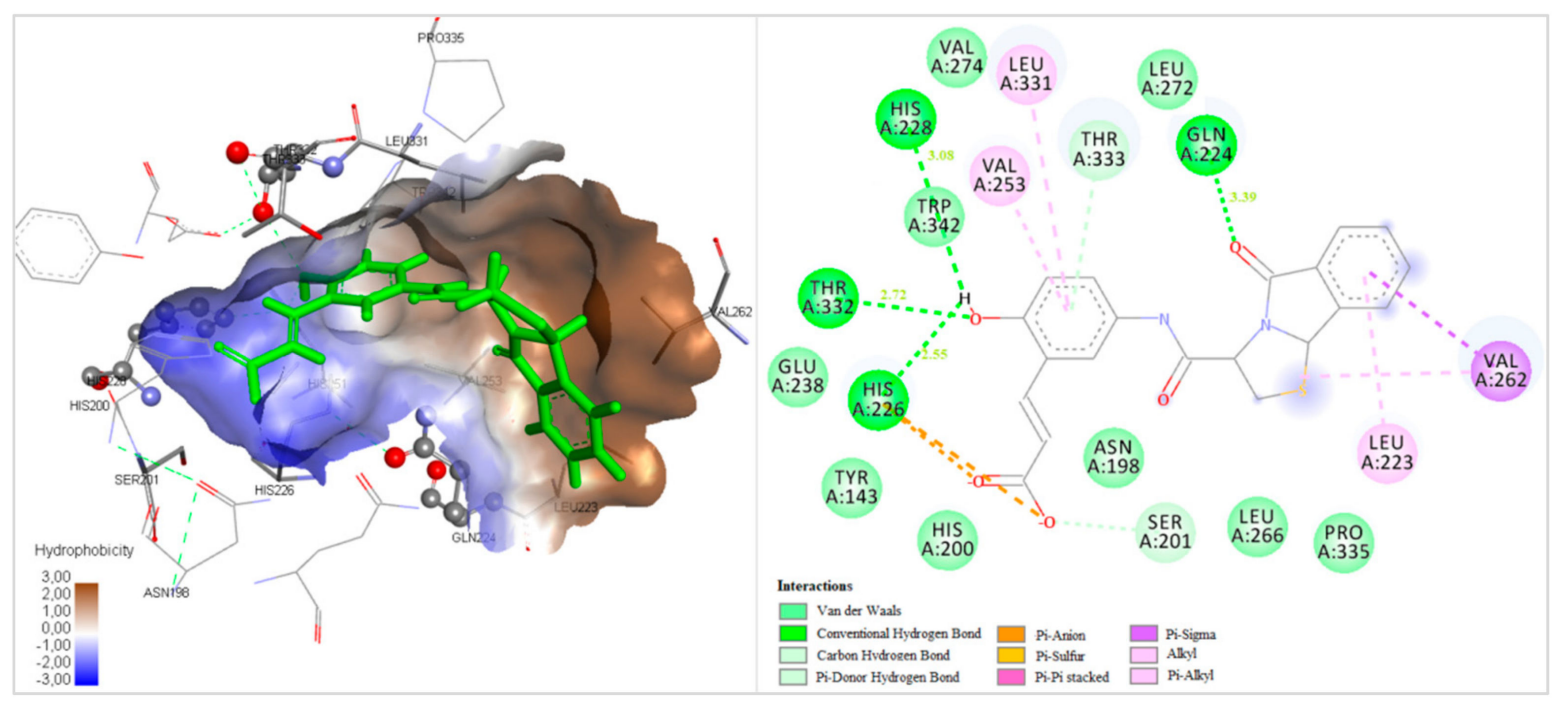
(**left**) Docked pose of **H11** in hCA IX active site; (**right**) 2D ligand interaction diagram for the docked ligand.

**Figure 3:**
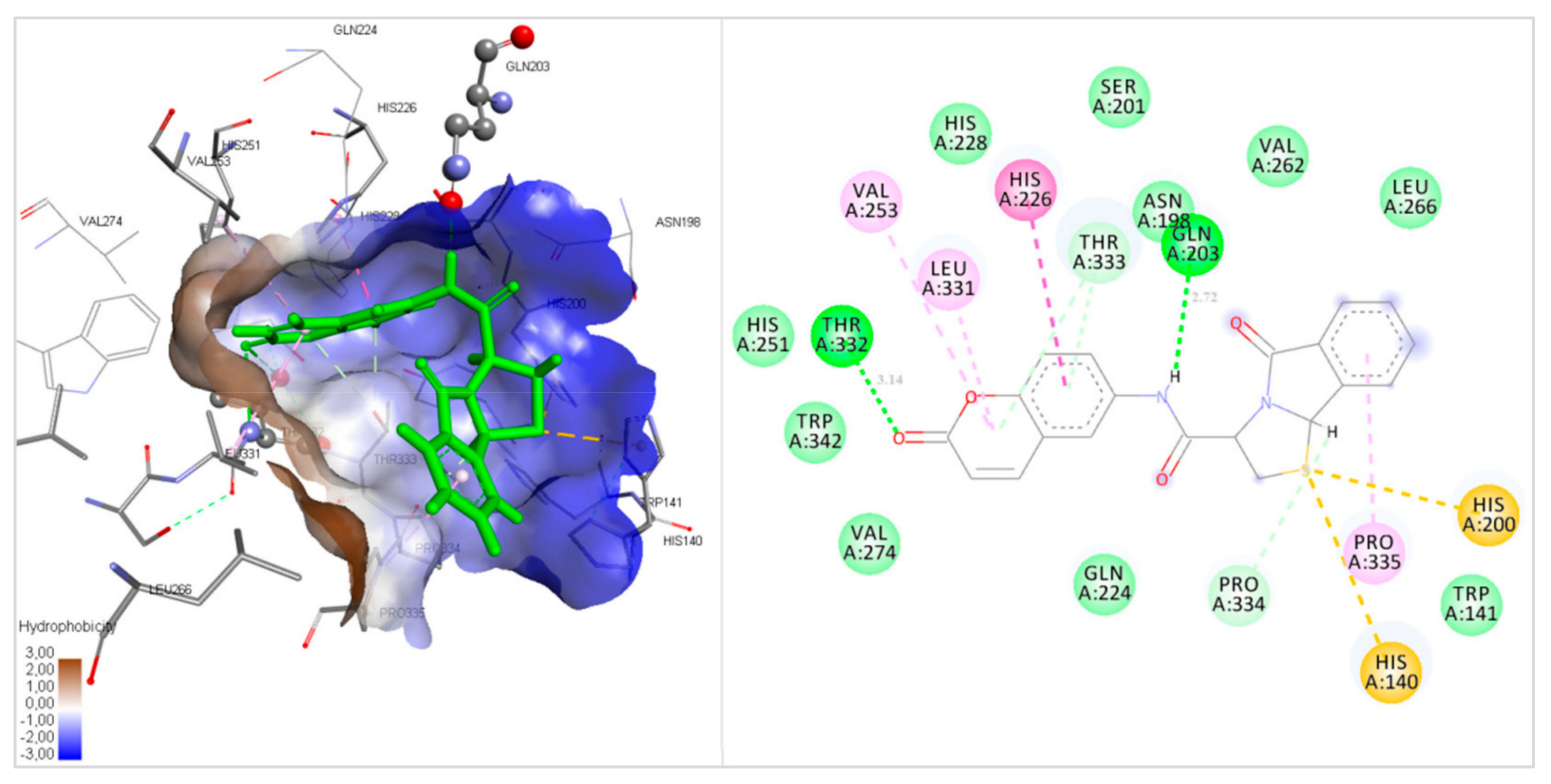
(**left**) Docked pose of **11** in hCA IX active site; (**right**) 2D ligand interaction diagram for the docked ligand.

Docking studies with hCA IX and XII showed that the free energy of binding of the compounds hydrolyzed species (**H**) is lower than that of the compounds themselves. As a result, we can indicate the hydrolyzed form of the compounds as the responsible for the CA inhibition. For hCA IX, the best docking score was predicted for **H11** which was also kinetically reported as the best isozyme inhibitor (Table 1). The docked poses of compound **11** and its hydrolyzed product (**H11**) are reported in Figure 2 and Figure 3. **H11** formed four hydrogen bonds with residues Gln224, His226, His228 and Thr332. The phenyl ring showed hydrophobic interactions with residues Tyr143, Asn198, Ser201, Val253, Leu331, and Thr333, while the fused rings interact hydrophobically with residues Leu223, Val262, Leu266, Leu272, and Pro335. In contrast, compound **11** forms only two hydrogen bonds within the enzyme binding site with residues Gln203 and Thr332 (Figure 3). This might explain the better scores reported by **H11** in comparison to **11**. 

**Figure 4:**
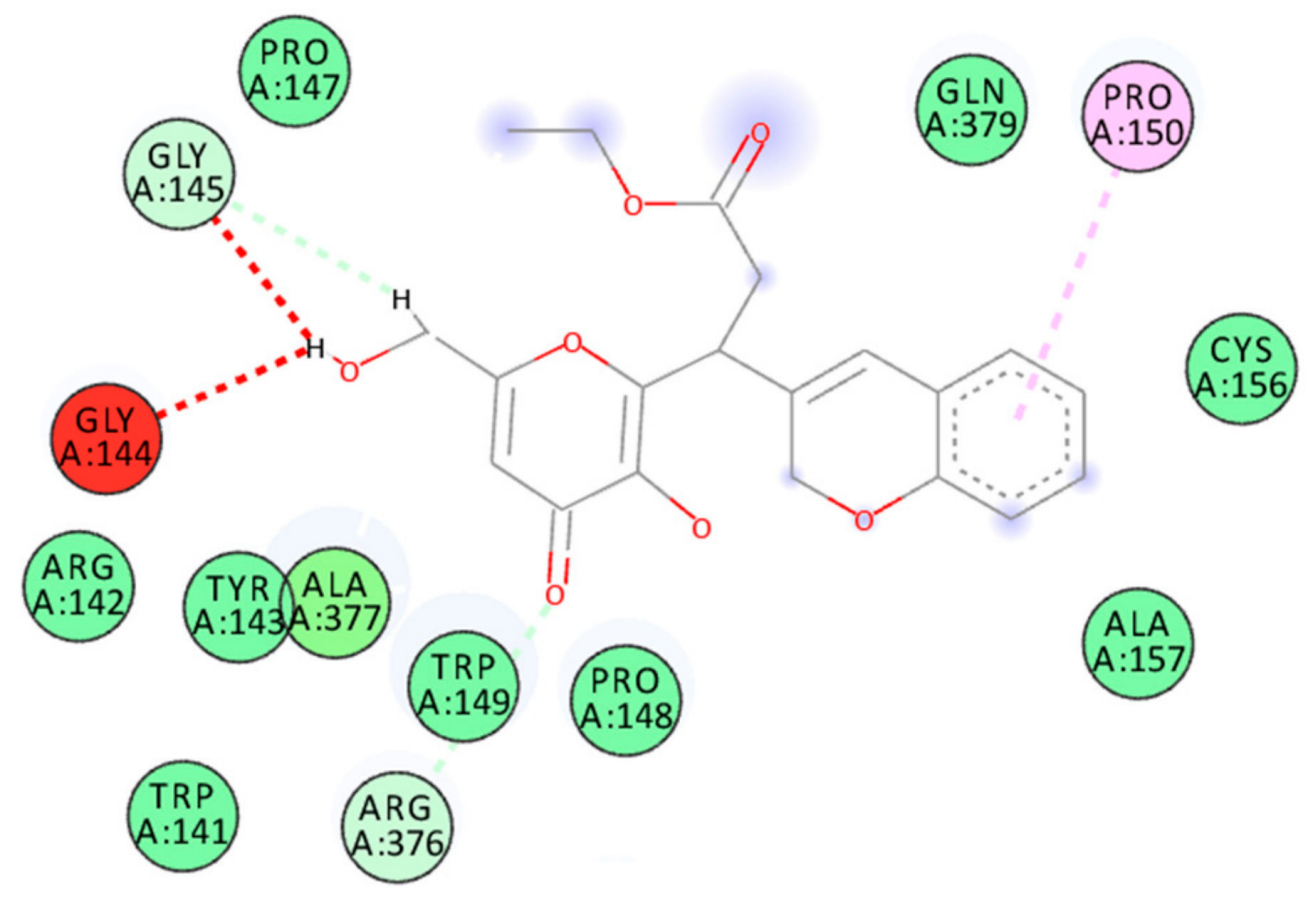
2D ligand interaction diagram for compound **4** docked in hCA IX active site.

Some repulsions taking place in the adduct of compound **4** within the isozyme binding site (red-colored residues Gly144, Gly145 in Figure 4) might account for its weaker hCA IX inhibition with respect to the other derivatives. These repulsive forces between the ligand and the active site residues likely do not allow the ligand to adopt the proper conformation into the cavity.

**Figure 5:**
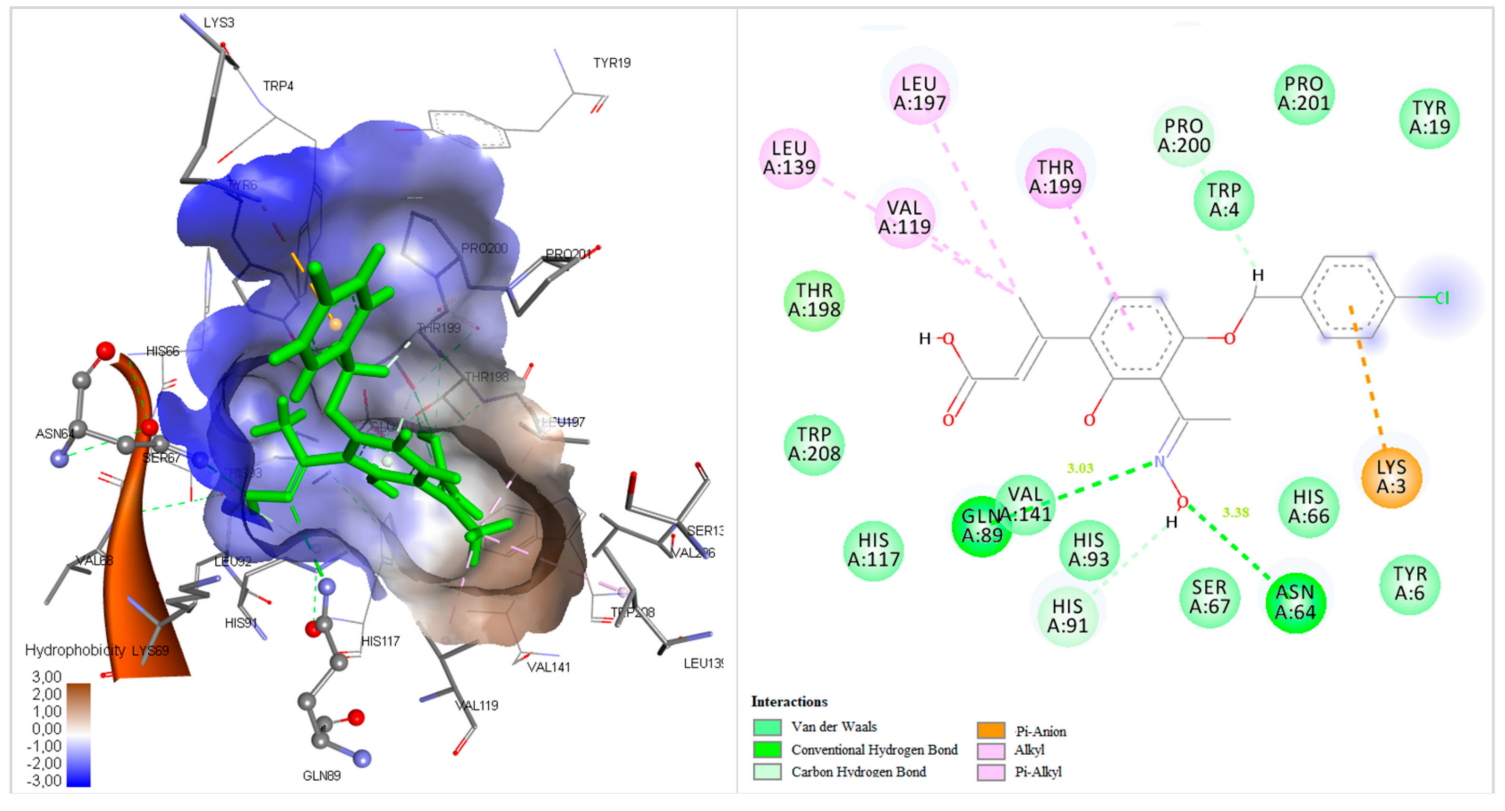
(**left**) Docked pose of **H1** in hCA IX active site; (**right**) 2D ligand interaction diagram for the docked ligand.

For hCA XII, the in vitro and docking results were in accordance as all compound showed similar docking scores. Nonetheless, **H1** showed a somewhat higher score compared to other derivatives (Table 2). According to the 2D ligand interaction figure (Figure 5), H**1** forms 2 H-bonding interactions with residues Asn64 and Gln89 of the target protein. It also forms hydrophobic interactions with residues Tyr6, His66, Ser67, His93, Val141, Tyr198, Val119, Leu197, Leu139 Thr199, and Pro200.

## 3. Materials and Methods 

### 3.1. Chemistry

Solvents, unless otherwise specified, were of analytical reagent grade or of the highest quality commercially available. Synthetic starting materials, reagents and solvents were purchased from InterBioscreen (Chernogolovka, Russia, https://www.ibscreen.com/) and Aldrich Chemie (Steinheimm, Germany). Melting points (°C) were determined with a Boetius apparatus (Dresden, Germany) without correction. ^1^H-NMR spectra of the newly synthesized compounds in DMSO-d_6_ solutions were recorded on a Bruker AC 300 instrument (Bruker, Karlsruhe, Germany) at 298 K. Chemical shift (δ) values for ^1^H-NMR spectra are reported in parts per million (ppm) with the solvent resonance as the internal standard. The TLC analysis was performed with Merck Silica Gel 60 F254 precoated plates, and each of the synthesized compounds showed a single spot.

Compounds **9** [34], **10** [35] were synthesized according to the literature procedures. Compounds **11**, **12** were purchased from InterBioscreen (Chernogolovka, Russia, https://www.ibscreen.com/).

#### 3.1.1. 7-((4-Chlorobenzyl)oxy)-8-(1-(hydroxyimino)ethyl)-4-methyl-2H-chromen-2-one (**1**)

To a stirred solution of 0.66 g (3.0 mmol) 8-acetyl-7-hydroxy-4-methylcoumarin [37] in 50 mL acetone was added 1.38 g (10 mmol) K_2_CO_3_ and 0.53 g (3.3 mmol) 4-chlorobenzyl chloride. Reaction mixture was stirred at 50–60 °C for 8 h, poured into 200 ml of water and acidified with HCl until pH 4-5. The residue was filtered off, dried, and crystallized from i-PrOH-H_2_O mixture afford title compound **1a**. Yield was 87%.

The mixture of 0.68 g (2.0 mmol) of 8-acetylcoumarin **1a**, 0.49 g (5.0 mmol) KOAc, 0.30 g (4.0 mmol) hydroxylamine hydrochloride was refluxed for 3 h. Then reaction mixture was evaporated in vacuo and the residue was purified by re-crystallization from MeOH–H_2_O (1:1) to afford pure product **1**. Yield was 66%, m.p. 208–210 °C. ^1^H-NMR (300 MHz, DMSO-d_6_) δ 2.11 (s, 3H, CH_3_), 2.42 (s, 3H, CH_3_), 5.23 (s, 2H, CH_2_), 6.11 (s, 1H, CH), 7.05 (d, *J* = 8.8 Hz, 1H, 2CH), 7.35 (d, *J* = 8.9 Hz, 2H), 7.41 (d, *J* = 8.9 Hz, 2Hz, 2H), 7.61 (d, *J* = 8.8 Hz, 1H, CH), 10.92 (s, 1H, OH); ^13^C-NMR (500 MHz, CDCl_3_**):** δ ppm 12.68, 20.11, 71.93, 111.92, 113.99, 114.83, 114.98, 128.72, 129.72(2C), 130.20 (2C), 132.88, 136.64, 149.57, 154.67, 156.83, 159.57, 161.50.

#### 3.1.2. 6’-((Dimethylamino)methyl)-7’-hydroxy-8’-methyl-2H,2’H-[3,4’-bichromene]-2,2’-dione (**2**)

Starting bicoumarin **2a** was obtained from (7-hydroxy-8-methylycoumarin-4-yl)acetic acid methyl ester and 2-hydroxybenzaldehyde using the procedure described for the product **7**.

To a stirred suspension of bicoumarin **2a** (2 mmol) in 10 mL of 1,4-dioxane was added 0.3 mL (2.2 mmol, 1.1 eq) of bis(N,N-dimethylamino)methane at 70 °C. The mixture was heated at 80 °C for 2 h. Then reaction mixture was evaporated in vacuo and the residue was purified by recrystallization from isopropanol-hexane. Yield was 83%, m.p. 239–241^o^C. ^1^H-NMR (300 MHz, DMSO-d_6_) δ 2.18 (s, 3H, CH_3_), 2.32 (s, 6H, N(CH_3_)_2_), 3.63 (s, 2H, CH_2_), 6.25 (s, 1H, CH), 6.80 (s, 1H, CH), 7.35–7.49 (m, 2H, 2CH), 7.56–7.70 (m, 2H, 2CH), 7.85 (s, 1H, CH); ^13^C-NMR (500 MHz, CDCl_3_): δ ppm 9.69, 43.66 (2C), 104.43, 106.55, 113.98, 119.93, 121.41, 121.56, 125.41, 128.56, 128.78, 129.62, 130.43, 135.55, 137.43, 140.27, 145.67, 153.55, 160.43, 161.48. 

#### 3.1.3. 7,7’,8’-Trihydroxy-2H,2’H-[3,4’-bichromene]-2,2’-dione (**3**)

A solution of (7’,8’-dihydroxycoumarin-4-yl)acetic acid methyl ester [38] (2 mmol), 2,4-dihydroxybenzaldehyde (2 mmol), and DBU (0.2 mmol) in abs. dioxane (10 ml) was stirred at 100–105 °C for 10 h. The solution was cooled, the solvent evaporated under reduced pressure, and the residue transferred into acidified water (200 ml). The precipitated crystals were filtered off, washed on the filter with water, dried, and recrystallized from a DMF–MeOH (1:3 mixture). Yield was 45%, m.p. > 270 °C. ^1^H-NMR (300 MHz, DMSO-d_6_) δ 6.23 (s, 1H, CH), 6.70–6.82 (m, 4H, 4CH), 7.58 (d, *J* = 7.8 Hz, 1H, CH), 8.08 (s, 1H, CH); ^13^C-NMR (500 MHz, CDCl_3_): δ ppm 101.71, 102.88, 111.88, 111.90, 117.13, 121.31, 123.87, 125.31, 129.97, 130.14, 132.14, 137.37, 146.10, 148.11, 155.37, 158.31, 159.38, 160.65.

#### 3.1.4. Ethyl 3-(2H-chromen-3-yl)-3-(3-hydroxy-6-(hydroxymethyl)-4-oxo-4H-pyran-2-yl) propanoate (**4**)

The mixture of 0.53 g (3.3 mmol) of 2H-chromene-3-carbaldehyde, 0.43 g (3 mmol) of kojic acid, 0.48 g (3.3 mmol) of Meldrum’s acid and 0.33 g (3.3 mmol) of triethylamine in 7 ml of EtOH was refluxed for 2 h. Then reaction mixture was evaporated in vacuo and the residue was purified by column chromatography (SiO_2_, EtOAc–hexane, 2:1) to afford pure product **4**. Yield was 68%, m.p. 146–148 °C. ^1^H-NMR (300 MHz, DMSO-d_6_) δ 1.18 (t, *J* = 7.1 Hz, 3H, CH_3_), 2.93 (d, *J* = 8.0 Hz, 2H, CH_2_), 4.02 (q, *J* = 7.1 Hz, 2H, CH_2_), 4.21 (t, *J* = 8.0 Hz, 1H, CH), 4.28 (s, 2H, CH_2_), 4.71 (s, 2H, CH_2_), 5.49 (br.s, 1H, OH), 6.27 (s, 1H, CH), 6.37 (s, 1H, CH), 6.68 (d, *J* = 8.0 Hz, 1H, CH), 6.79 (m, 1H, CH), 7.02 (m, 2H, 2CH), 8.89 (br.s, 1H, OH); ^13^C-NMR (500 MHz, CDCl_3_): δ ppm 14.57, 27.21, 38.21, 57.88, 62.57, 63.52, 112.82, 114.12, 114.52, 115.42, 122.52, 126.41, 127.30, 140.25, 142.21, 157.25, 158.80, 173.69, 177.27, 181.63.

#### 3.1.5. General Procedure for The Synthesis of Compounds **5***–***8**

The mixture of 0.46 g (3 mmol) of 2,4-dihydroxyacetophenone, corresponding aldehyde (3.3 mmol), 0.58 g (4 mmol) of Meldrum’s acid, and 0.45 g (4.5 mmol) of triethylamine in 7 ml of MeOH was refluxed for 3 h. Then the reaction mixture was evaporated in vacuo and the residue was refluxed in 7 ml of AcOH for 4 h. Then reaction mixture was evaporated in vacuo and the residue was purified by column chromatography (SiO_2_, EtOAc–hexane, 1:1) to afford pure products **5***–***8**.

#### 3.1.6. 6-Acetyl-4-(3-(2-(2,3-dihydrobenzofuran-5-yl)ethoxy)-4-methoxyphenyl)-5-hydroxychroman-2-one (**5**)

Yield was 52%, m.p. 124–126 ^°^C. ^1^H-NMR (300 MHz, DMSO-d_6_) δ 2.64 (s, 3H, CH_3_), 2.94 (m, 3H, CH_2_), 3.15 (m, 3H, CH_2_), 3.69 (s, 3H, CH_3_), 4.04 (m, 2H, CH_2_), 4.53 (m, 3H, CH_2_ + CH), 6.40 (m, 1H, CH), 6.68 (d, *J* = 8.8 Hz, 1H, CH), 6.83 (m, 3H, 3CH), 6.99 (m, 1H, CH), 7.17 (m, 1H, CH), 8.02 (d, *J* = 8.8 Hz, 1H, CH), 12.96 (s, 1H, OH); ^13^C-NMR (500 MHz, CDCl_3_): δ ppm 26.69, 29.62,34.66, 35.55, 36.43, 55.67, 67.48, 81.41, 111.43,113.48, 114.55, 114.69, 119.93, 121.41, 125.62, 128.52, 128.78, 129.62, 130.77, 138.45, 148.87, 153.88, 155.81, 160.48, 167.52, 202.67.

#### 3.1.7. 6-Acetyl-5-hydroxy-4-(4-hydroxy-3-methoxyphenyl)chroman-2-one (**6**)

Yield was 43%, m.p. 159–161 ^o^C. ^1^H-NMR (300 MHz, DMSO-d_6_) δ 2.63 (s, 3H, CH_3_), 2.94 (dd, *J* = 1.8 Hz, *J* = 15.9 Hz, 1H, CH_2_), 3.16 (dd, *J* = 7.0 Hz, *J*= 15.9 Hz, 1H, CH_2_), 3.75 (s, 3H, CH_3_), 4.57 (dd, *J* = 1.8 Hz, *J* = 7.0 Hz, 1H, CH), 6.37 (d, *J* = 8.2 Hz, 1H, CH), 6.58–6.77 (m, 3H, 3CH), 7.93 (d, *J* = 8.9 Hz, 1H, CH), 8.54 (br.s, 1H, OH), 12.94 (s, 1H, OH); ^13^C-NMR (500 MHz, CDCl_3_): δ ppm 26.29, 34.62, 36.43, 56.66, 114.16, 114.69, 115.62, 116.45, 119.93, 121.51, 129.68, 138.41, 146.77, 148.87, 155.52, 161.48, 167.78, 202.43.

#### 3.1.8. 6-Acetyl-5-hydroxy-4-(2-(pyridin-2-ylmethoxy)phenyl)chroman-2-one (**7**)

Yield was 63%, m.p. 213–215 ^o^C. ^1^H-NMR (300 MHz, DMSO-d_6_) δ 2.63 (s, 3H, CH_3_), 2.98 (dd, *J* = 2.0 Hz, *J* = 16.1 Hz, 1H, CH_2_), 3.20 (dd, *J* = 7.7 Hz, *J* = 16.1 Hz, 1H, CH_2_), 4.97 (dd, *J* = 2.0 Hz, *J* = 7.7 Hz, 1H, CH), 5.25 (s, 2H, CH_2_), 6.77 (m, 3H, 3CH), 6.96 (m, 1H, CH), 7.10 (m, 1H, CH), 7.16 (m, 1H, CH), 7.29 (m, 1H, CH), 7.77 (m, 1H, CH), 7.96 (d, *J* = 8.8 Hz, 1H, CH), 8.56 (m, 1H, CH), 12.90 (s, 1H, OH); ^13^C-NMR (500 MHz, CDCl_3_): δ ppm 26.49, 30.59, 34.75, 70.36, 108,13, 112.25, 112.399, 116.37, 121.13, 121.25, 133.72, 128.33, 128.36, 128.87, 131.29, 137.10, 148.98, 155.43, 156.95, 157.65, 131.22, 166.44, 203.35.

#### 3.1.9. 3-(6-Acetyl-5-hydroxy-2-oxochroman-4-yl)-6-methoxy-4H-chromen-4-one (**8**)

Yield was 58%, m.p. 197–199 ^o^C. ^1^H-NMR (300 MHz, DMSO-d_6_) δ 2.59 (s, 3H, CH_3_), 2.81 (d, *J* = 16.7 Hz, 1H, CH_2_), 3.21 (dd, *J* = 8.8 Hz, *J* = 16.7 Hz, 1H, CH_2_), 3.84 (s, 3H, CH_3_), 4.42 (d, *J* = 8.8 Hz, 1H, CH), 6.66 (d, *J* = 8.9 Hz, 1H, CH), 7.29 (m, 1H, CH), 7.36 (m, 1H, CH), 7.53 (m, 1H, CH), 7.88 (d, *J* = 8.9 Hz, 1H, CH), 8.07 (s, 1H, CH), 13.03 (s, 1H, OH); ^13^C-NMR (500 MHz, CDCl_3_): δ ppm 26.49, 30.36, 34.73, 56.95, 105.15, 114.39, 116.72, 119.12, 119.25, 122.87, 123.36, 123.83, 127.43, 148.93, 150.13, 131.65, 156.91, 159.22, 166.41, 183.33, 203.36.

### 3.2. CA Inhibition

An Applied Photophysics stopped-flow instrument was used for assaying the CA-catalysed CO_2_ hydration activity [36]. Phenol red (at a concentration of 0.2 mM) was used as an indicator, working at the absorbance maximum of 557 nm, with 20 mM Hepes (pH 7.5) as buffer, and 20 mM Na_2_SO_4_ (for maintaining constant the ionic strength), following the initial rates of the CA-catalysed CO_2_ hydration reaction for a period of 10–100 s. The CO_2_ concentrations ranged from 1.7 to 17 mM for the determination of the kinetic parameters and inhibition constants. For each inhibitor, at least six traces of the initial 5–10% of the reaction have been used for determining the initial velocity. The uncatalyzed rates were determined in the same manner and subtracted from the total observed rates. Stock solutions of inhibitor (0.1 mM) were prepared in distilled-deionised water and dilutions up to 0.1 nM were done thereafter with the assay buffer. Inhibitor and enzyme solutions were preincubated together for 6 h at room temperature prior to assay, in order to allow for the formation of the E–I complex (coumarins act as pro-drug inhibitors). The inhibition constants were obtained by nonlinear least-squares methods using PRISM 3 and the Cheng-Prusoff equation, as reported earlier [39], and represent the mean from at least three different determinations. All CA isoforms were recombinant ones obtained in-house, as reported earlier [40,41,42].

### 3.3. Molecular Modeling 

Docking calculations were carried out using the AutoDock 4.2 software. The free energy of binding (ΔG) of both CA IX and XII complexes with the compounds was generated using this molecular docking program. The crystal structures of CA IX (PDB code 5DVX) and CA XII (PDB code 5MSA) were taken from the Protein Data Bank [43,44]. For the enzymes’ preparation, polar hydrogens were added; Kollman United Atom charges and atomic salvation parameters were assigned. For ligands preparation, Gasteiger partial charges were added, non-polar hydrogen atoms were merged, and rotatable bonds were defined. The three-dimensional structures of all the compounds were assembled using Chem3Dultra 12.0 software. The grid size was set to 50 × 50 × 50 xyz points with grid spacing of 0.375 Å. The grid centers were calculated for CA IX (X = 5.741, Y = −15.751 and Z = 8.657) and for CA XII (X = 24.159, Y = 9.619 and Z = 21.1). All parameters used in docking were default. A primary blind docking was performed to find the favored binding sites of the ligand to the receptor. The Lamarckian genetic algorithm was applied for minimization using default parameters. The number of docking runs was 100. After docking, the 100 solutions were clustered into groups with RMS lower than 1.0 Ε. The discovery studio 2017 R2 silent (BIOVIA, San Diego, CA, USA) was used for the virtualization of the resulting poses and potential interactions.

## 4. Conclusions

We reported here the synthesis, characterization, and kinetic/in silico evaluation of a set of new coumarin/dihydrocoumarin derivatives as inhibitors of human CA. In detail, we investigated the compounds for the inhibition of the cytosolic human isoforms hCA I and II and the transmembrane, tumor-associated hCA IX and hCA XII that are validated target for anticancer intervention. Two compounds were identified which showed potent inhibitory activity against hCA IX with K_I_s of 9.4 and 25.7 nM, thus being more active or equipotent with the reference drug acetazolamide. A computational assessment was performed to gain insights on the binding mode of this class of compounds within the active site of hCA IX and XII. Docking studies with hCA IX and XII revealed that the free energy of binding of the hydrolyzed species (**H**) is lower than that of the compounds themselves indicating that probably, the hydrolyzed form is responsible for the CA inhibition. For hCA IX, the best docking score was predicted for **H11,** which was also kinetically reported as the best isozyme inhibitor.

## Figures and Tables

**Table 1:** Inhibition data of hCA I, hCA II, hCA IX, and hCA XII with compounds **1**–**12** and the standard inhibitor acetazolamide (AAZ) by a Stopped Flow CO_2_ Hydrase Assay [36].

Cmpd	K_I_^*^ (nM)			
	hCA II	hCA II	hCA IX	hCAXII
**1**	>10,000	>10,000	49.3	558.1
**2**	>10,000	>10,000	85.6	>10,000
**3**	>10,000	>10,000	132.6	>10,000
**4**	>10,000	>10,000	243.1	466.7
**5**	>10,000	>10,000	171.6	>10,000
**6**	>10,000	>10,000	188.6	>10,000
**7**	>10,000	>10,000	138.6	>10,000
**8**	>10,000	>10,000	174.8	>10,000
**9**	>10,000	>10,000	171.2	>10,000
**10**	>10,000	>10,000	25.7	603.8
**11**	>10,000	>10,000	9.4	590.9
**12**	>10,000	>10,000	240.9	432.1
**AAZ**	250	12	25	5.7

* Mean from 3 different assays, by a stopped flow technique (errors were in the range of ± 5–10 % of the reported values).

**Table 2:** Molecular docking binding affinities for compounds **1**–**12** and their hydrolyzed (**H**) species.

	Estimated Binding Energy (kcal/mol)		Binding Affinity Score	I-H	Residues CA IX
	CA-XII	CA-IX			
**1**	−5.13	−7.03	−26.49	1	Thr332
**H1**	−6.89	−8.16	−27.25	2	Gln224, Thr333
**2**	−1.24	−6.80	−23.37	1	Gln203
**H2**	−3.85	−7.55	−25.12	2	Gln224, Thr332
**3**	-	−6.33	−21.13	1	Thr332
**H3**	−3.21	−6.74	−21.42	1	Thr333
**4**	−3.11	−5.13	−15.96	-	-
**5**	-	−6.11	−20.09	1	Thr332
**H5**	-	−6.77	−20.83	1	His228
**6**	-	−5.81	−17.26	-	-
**H6**	-	−6.05	−18.73	1	Thr333
**7**	-	−6.24	−21.05	1	Thr332
**H7**	−2.57	−6.72	−21.41	1	Thr333
**8**	-	−6.03	−19.26	-	-
**8**	−1.22	−6.15	−19.58	1	Thr333
**5 9**	-	−6.10	−20.27	1	Gln203
**H9**	-	−6.79	−20.97	1	Thr333
**10**	−3.49	−8.22	−27.34	1	Thr332
**H10**	−5.88	−10.03	−29.78	2	Thr332, Thr333
**11**	−5.36	−9.61	−29.14	2	Gln203, Thr332
**H11**	−5.41	−13.25	−35.41	4	Gln224, His226, His228, Thr332
**12**	−3.18	−5.28	−16.47	-	-

## References

[1] Jain P.K., Joshi H. (2012). Coumarin: Chemical and Pharmacological Profile. J. Appl. Pharm. Sci..

[2] Abdelhafez O.M., Amin K.M., Batran R.Z., Maher T.J., Nada S.A., Sethumadhavan S. (2010). Synthesis, anticoagulant and PIVKA-II induced by new 4-hydroxycoumarin derivatives. Bioorg. Med. Chem..

[3] Saidu N.E., Valente S., Bana E., Kirsch G., Bagrel D., Montenarh M. (2012). Coumarin polysulfides inhibit cell growth and induce apoptosis in HCT116 colon cancer cells. Bioorg. Med. Chem..

[4] Kim S.N., Kim N.H., Park Y.S., Kim H., Lee S., Wang Q., Kim Y.K. (2009). 7-Diethylamino-3(2’-benzoxazolyl)-coumarin is a novel microtube inhibitor with antimitotic activity in multidrug resistant cancer cells. Biochem. Pharmacol..

[5] Lin M.H., Cheng C.H., Chen K.C., Lee W.T., Wang Y.F., Xiao C.Q., Lin C.W. (2014). Inhibition of ros-independent jink-activation-mediated apoptosis by a novel coumarin derivative, DMAK in human colon cancer cells. Chem-Biol. Interac..

[6] Li Z., Hu J., Sun M., Ji H., Chu S., Liu G., Chen N. (2012). Anti-inflammatory effect of IMMLG5521, a coumarin derivative, on Sephades-induced lung inflammation in rats. Int. Immunopharmacol..

[7] Togna A.R., Firuzi O., Latina V., Parmar V.S., Prasad A.K., Salemme A. (2014). 4-Methylcoumarin derivatives with anti-inflammatory effects on activated microglial cells. Biol. Pharm. Bull..

[8] Supuran C.T. (2017). Advances in structure-based drug discovery of carbonic anhydrase inhibitors. Expert Opin. Drug Discov..

[9] Supuran C.T. (2016). How many carbonic anhydrase inhibition mechanisms exist?. J. Enzyme Inhib. Med. Chem..

[10] Batra N., Batra S., Pareek A., Nagori B.P. (2012). Diverse harmacological activities of 3-substituted coumarins. Int. Res. J. Pharm..

[11] Khan K.M., Saify Z.S., Khan M.Z., Choudhary I.M., Perveen S., Chohan Z.H., Supuran C.T. (2004). Synthesis ofcoumarin derivatives with cytotoxic, antibacterial and antifungal activity. J. Enzym. Inhib. Med. Chem..

[12] Brooker N.L., Kuzimichev Y., Laas J., Pavlis R. (2007). Evaluation of coumarin derivatives as anti-fungal agents against soil-borne fungal pathogens. Commun. Agric. Appl. Biol. Sci..

[13] Han J., Sun L., Huang X., Li Z., Zhang C., Qian H., Huang W. (2014). Novel coumarin modified GLP-1derivatives with enhanced plasma stability and prolonged in vivo glucose -lowering ability. Br. J. Pharmacol..

[14] Yang J., Liu G.Y., Dai F., Cao X.Y., Kang Y.F., Hu L.M., Tang J.J., Li X.Z., Li Y., Jin X.L. (2011). Synthesis and biological evaluation of hydroxylated 3-phenylcoumarins as antioxidants and antiproliferative agents. Bioorg. Med. Chem. Lett..

[15] Anand P., Singh B., Singh N. (2012). Areview on coumarins as acetlcholinesterase inhibitors for Alzheimer’s disease. Bioorg. Med. Chem..

[16] Vina D., Matos M.L., Yanez M., Santana L., Uriarte E. (2012). 3-Substituted coumarins as dual inhibitors of AChE and MAO for the treatment of Alzheimer’s disease. MedChemCom..

[17] Xie S.-S., Wang X.-B., Li J.-Y., Yang L., Kong L.-Y. (2013). Design, synthesis and evaluation of novel tacrine-coumarin hubrids as multifunctional cholinesterase inhibitors against Alzheimer’s disease. Eur. J. Med. Chem..

[18] Huang M., Xie S.-S., Jiang N., Lan J.-S., Kong L.-Y., Wang X.-B. (2015). Multifunctional coumarin derivatives: Monoamine oxidase B (MAO-B) inhibition, anti-β-amyloid (Aβ) aggregation and metal chelation properties against Alzheimer’s disease. Bioorg. Med. Chem. Lett..

[19] Al-Majedy Y., Al-Amiery A., Kadhum A.A., Bakar M.A. (2017). Antioxidant activity of coumarins. Sys. Rev. Pharm..

[20] Kadhum A.A.H., Al-Amiery A.A., Musa A.Y., Mohamad A.B. (2011). The antioxidant activity of new coumarin derivatives. Int. J. Mol. Sci..

[21] Sashidhara K.V., Kumar A., Kumar M., Srivastava A., Puri A. (2010). Synthesis and antihyperlipidemic activity of novel coumarin bisindole derivatives. Bioorg. Med. Chem. Lett..

[22] Jalal S., Chand K., Kathuria A., Singh P., Priya N., Gupta B., Raj H.G., Sharma S.K. (2012). Calreticulin transacetylase: A novel enzyme-mediated protein acetylation by acetoxy derivativesof 3-alkyl-4-methylcoumarins. Bioorg. Chem..

[23] Lee J.H., Kim Y.G., Cho H.S., Ryu S.Y., Cho M.H., Lee J. (2014). Coumarins reduce biofilm formation and the virulence of Escherichia coli O157:H7. Phytomedicine.

[24] Mandlik V., Patil S., Bopanna R., Basu S., Singh S. (2016). Biological Activity of Coumarin Derivatives as Anti-Leishmanial Agents. Plos ONE.

[25] Melis C., Distinto S., Bianco G., Meleddu R., Cottiglia F., Fois B., Taverna D., Angius R., Alcaro S., Ortuso F. (2018). Targeting Tumor Associated Carbonic Anhydrases IX and XII: Highly Isozyme Selective Coumarin and Psoralen Inhibitors. ACS Med. Chem. Lett..

[26] Narayanaswamy V.K., Gleiser R.M., Kasumbwe K., Aldhubiab B.E., Attimarad M.V., Odhav B. (2014). Evaluation of Halogenated Coumarins for Antimosquito Properties. Sci. World J..

[27] Maresca A., Temperini C., Vu H., Pham N.B., Poulsen S.A., Scorrafava A., Quinn R.J., Supuran C.T. (2009). Non-zinc mediated inhibition of carbonic anhydrase; coumarins are a new class of suicide inhibitors. J. Am. Chem. Soc..

[28] Bonneau A., Maresca A., Winum J.-V., Supuran C.T. (2013). Metronidazole –coumain conjugates and 3-cyano-7-hydroxy-coumarin act as isoform-selective carbonic anhydrase inhibitor. J. Enz. Inhib. Med. Chem..

[29] Küçükbay F.Z., Küçükbay H., Tanc M., Supuran C.T. (2016). Synthesis and carbonic anhydrase inhibitory properties of amino acid-coumarin/quinolinone conjugates incorporating glycine, alanine and phenylalanine moieties. J. Enzyme. Inhib. Med. Chem..

[30] Maresca A., Supuran C.T. (2010). Coumarins incorporating hydroxy- and chloro-moieties selectively inhibit the transmembrane, tumor-associated carbonic anhydrase isoforms IX and XII over the cytosolic ones I and II. Bioorg. Med. Chem. Lett..

[31] Maresca A., Scozzafava A., Supuran C.T. (2010). 7,8-disubstituted- but not 6,7-disubstituted coumarins selectively inhibit the transmembrane, tumor-associated carbonic anhydrase isoforms IX and XII over the cytosolic ones I and II in the low nanomolar/subnanomolar range. Bioorg. Med. Chem. Lett..

[32] Supuran C.T. (2012). Structure-based drug discovery of carbonic anhydrase inhibitors. J. Enzym. Inhib. Med. Chem..

[33] Nocentini A., Supuran C.T. (2019). Advances in the structural annotation of human carbonic anhydrases and impact on future drug discovery. Expert Opin. Drug. Discov..

[34] Khilya V.P., Kovalev S.V., Miroshnichenko N.S., Turov A.V. (1998). Synthesis and spectral properties of 3-furyl-4-hydroxycoumarins. Chem. Nat. Compd..

[35] Szabo V., Grishko L.G., Borbei S., Khilya V.P. (1975). Chemistry of heteroanalogs of isoflavones. Chem. Heterocycl. Compd..

[36] Khalifah R.G. (1971). The carbon dioxide hydration activity of carbonic anhydrase. I. Stop-flow kinetic studies on the native human isoenzymes B and C. J. Biol. Chem..

[37] Bondarenko S.P., Frasinyuk M.S., Khilya V.P. (2003). Synthesis of Pseudobaptigenin Analogs. Chem. Nat. Compd..

[38] Galayev O., Garazd Y., Garazd M., Lesyk R. (2015). Synthesis and anticancer activity of 6-heteroarylcoumarins. Synthesis and anticancer activity of 6-heteroarylcoumarins. Eur. J. Med. Chem..

[39] Entezari Heravi Y., Bua S., Nocentini A., Del Prete S., Saboury A.A., Sereshti H., Capasso C., Gratteri P., Supuran C.T. (2017). Inhibition of Malassezia globosa carbonic anhydrase with phenols. Bioorg. Med. Chem..

[40] Nocentini A., Carta F., Tanc M., Selleri S., Supuran C.T., Bazzicalupi C., Gratteri P. (2018). Deciphering the Mechanism of Human Carbonic Anhydrases Inhibition with Sulfocoumarins: Computational and Experimental Studies. Chemistry.

[41] Nocentini A., Gratteri P., Supuran C.T. (2019). Phosphorus versus Sulfur: Discovery of Benzenephosphonamidates as Versatile Sulfonamide-Mimic Chemotypes Acting as Carbonic Anhydrase Inhibitors. Chemistry.

[42] Nocentini A., Trallori E., Singh S., Lomelino C.L., Bartolucci G., Di Cesare Mannelli L., Ghelardini C., McKenna R., Gratteri P., Supuran C.T. (2018). 4-Hydroxy-3-nitro-5-ureido-benzenesulfonamides Selectively Target the Tumor-Associated Carbonic Anhydrase Isoforms IX and XII Showing Hypoxia-Enhanced Antiproliferative Profiles. J. Med. Chem..

[43] Mahon B.P., Bhatt A., Socorro L., Driscoll J.M., Okoh C., Lomelino C.L., Mboge M.Y., Kurian J.J., Tu C., Agbandje-McKenna M. (2016). The Structure of Carbonic Anhydrase IX Is Adapted for Low-pH Catalysis. Biochemistry.

[44] Whittington D.A., Waheed A., Ulmasov B., Shah G.N., Grubb J.H., Sly W.S., Christianson D.W. (2001). Crystal structure of the dimeric extracellular domain of human carbonic anhydrase XII, a bitopic membrane protein overexpressed in certain cancer tumor cells. Proc. Natl. Acad. Sci. USA.

